# Toward Exosome-Based Neuronal Diagnostic Devices

**DOI:** 10.3390/mi9120634

**Published:** 2018-11-29

**Authors:** Yong Kyoung Yoo, Junwoo Lee, Hyungsuk Kim, Kyo Seon Hwang, Dae Sung Yoon, Jeong Hoon Lee

**Affiliations:** 1 Department of Electrical Engineering, Kwangwoon University, 447-1 Wolgye, Nowon, Seoul 01897, Korea; yongkyoung0108@gmail.com (Y.K.Y.); mindsjw@gmail.com (J.L.); hskim@kw.ac.kr (H.K.); 2 Department of Clinical Pharmacology and Therapeutics, College of Medicine, Kyung Hee University, Seoul 02447, Korea; k.hwang@khu.ac.kr; 3 School of Biomedical Engineering, Korea University, Seoul 02841, Korea

**Keywords:** Alzheimer’s disease, diagnostics, exosome, Parkinson’s disease

## Abstract

Targeting exosome for liquid biopsy has gained significant attention for its diagnostic and therapeutic potential. For detecting neuronal disease diagnosis such as Alzheimer’s disease (AD), the main technique for identifying AD still relies on positron-emission tomography (PET) imaging to detect the presence of amyloid-β (Aβ). While the detection of Aβ in cerebrospinal fluid has also been suggested as a marker for AD, the lack of quantitative measurements has compromised existing assays. In cerebrospinal fluid, in addition to Aβ, T-Tau, and P-Tau, alpha-synuclein has been considered a biomarker of neurodegeneration. This review suggests that and explains how the exosome can be used as a neuronal diagnostic component. To this end, we summarize current progress in exosome preparation/isolation and quantification techniques and comment on the outlooks for neuronal exosome-based diagnostic techniques.

## 1. Introduction

Alzheimer’s disease (AD) and Parkinson’s disease (PD) are the most prevalent age-related neurodegenerative disorders that result from synaptic degeneration and nerve cell death [1]. Dementia is a general mental disorder, characterized by memory impairment and loss of judgment, and around 50% of all patients with dementia have comorbid AD.

AD currently affects around 5.5 million adults in the United States and this figure is expected to increase to 13.8 million by 2050 [2]. It has been estimated that new AD diagnoses were made every 66 s in 2016, an estimate which has reached one in every 33 s in 2050 [3]. In addition, it is estimated that, in 2014, 29.3 per 100,000 people of the population died from AD in the United States. Furthermore, more than 15 million families and caregivers in the United States dedicated about 18.2 billion hours of care to patients with AD or dementia in 2016, which has been estimated to have cost over $230 billion [3]. In addition, the total cost of dementia-related health care, long-term care, and hospice services was estimated to have been $259 billion in 2017, and the social costs of dementia due to AD are only increasing [3].

The early stage detection and diagnosis of AD plays a key role in patient care, since early detection allows patients to take preventative measures before irreversible brain damage occurs [4,5,6]. The Mini-Mental State Examination (MMSE) and the Logical Memory (LM) test are used to measure cognitive status in patients with AD [7]. Generally, biomarkers, such as amyloid-β (Aβ), deposition in the brain can be assessed using neuroimaging methods (i.e., MRI and PET) or cerebrospinal fluid (CSF) analysis [8,9,10,11].

The pathogenesis of AD has been reported to be potentially driven by excessive production and deposition of Aβ protein. Such abnormal Aβ levels can typically be detected in biofluids, such as CSF. It has been reported that continuously monitoring Aβ levels could facilitate early AD diagnosis and treatment before the onset of AD symptoms [11,12]. Nakamura et al. recently demonstrated blood-based clinical validation using plasma-based Aβ had both cost–benefit and scalability advantages [13].

**Figure 1:**
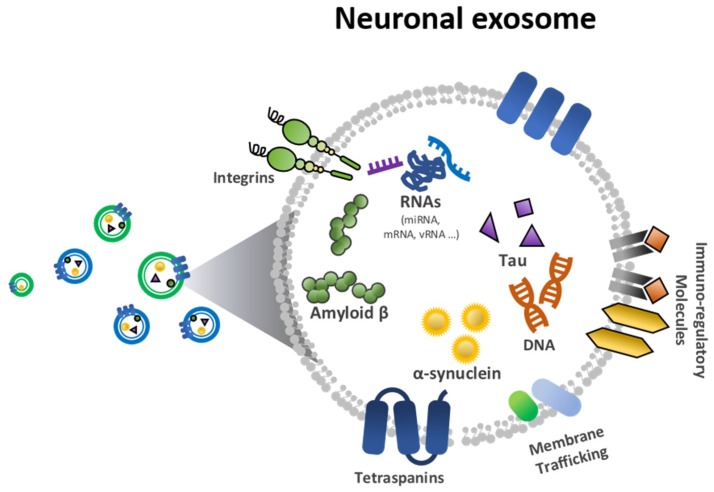
Biological elements in a neuronal exosome, including amyloid-β (Aβ), tau and α-synuclein.

With regard to clinical utility, much effort has been made to validate methods for monitoring neurodegenerative disorders. The exosome has been highlighted for its potential in early stage diagnostics. Nanoscale exosomes (30–150 nm) have been studied to assess proteomic and genetic information about disease diagnostics [14,15]. In particular, neuronal exosomes, as shown in Figure 1, are good candidates for detecting neurodegenerative biomarkers [16,17,18]. Among neurodegenerative biomarkers, Aβ and tau have been considered protein markers of AD and α-synuclein a biomarker of PD (Figure 1).

**Figure 2:**
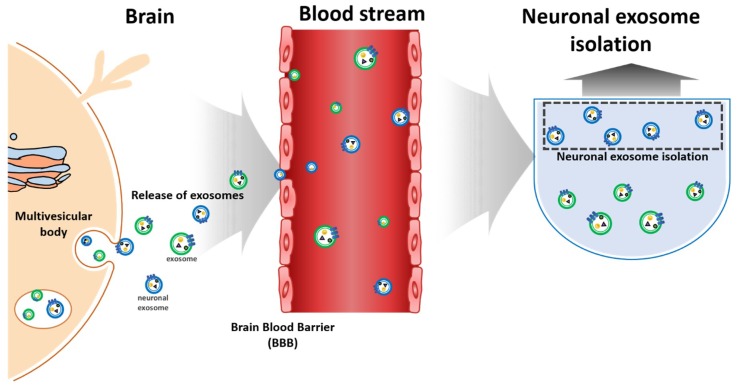
Schemes of neuronal exosomes isolation/detection from blood.

Typically, neuronal exosomes are collected from human CSF, and the protein markers from a neuronal exosome, i.e., Aβ, tau and α-synuclein, can be extracted from this CSF (Figure 2) [19,20,21,22,23,24,25]. While sample protocols from CSF is well-established, it does present several risks, including pain and leaks that can cause infection, nerve damage, and headaches. The invasive nature of CSF collection means that blood-based detection might be the simplest and most powerful method [26,27]. Since neuronal exosomes can penetrate the blood-brain-barrier, they can be identified in the blood samples, as shown in Figure 2 [28,29].

To diagnose AD and PD using neuronal exosomes in the blood, exosomes must be isolated from the blood. Because of the paucity of biomarker studies in peripheral blood, it is difficult to link biomarker levels with brain pathology due to the relative uncertainty of their tissue of origin [30]. AD and PD diagnoses are thus limited by the unclear origin of exosomes in blood. In contrast, neuronal exosomes can be used to assess for biomarkers of AD and PD since they are known to originate from brain tissue [31]. Neuronal exosomes are thus not only possible, but advantageous, to use instead of exosomes for the identification of AD and PD biomarkers. There are many ways to implement this, and several studies have investigated how best to increase the purity and yield of exosome separations [32,33]. The next step is to separate neuronal exosomes from the isolated exosomes. While many techniques on how to separate exosomes from the blood have been reported, only one technique has been reported concerning the separation of neuronal exosomes from isolated exosomes, using a specific protein presented on the surface of the neuronal exosome [34]. Generally, the amount of exosome in the blood is not enough for a general-purpose ELISA; thus, new techniques that increase the yield and purity of both exosomes and neuronal exosomes are required [35].

## 2. Exosome Preparation/Isolation Methods

### 2.1. Differential Ultracentrifugation

**Figure 3:**
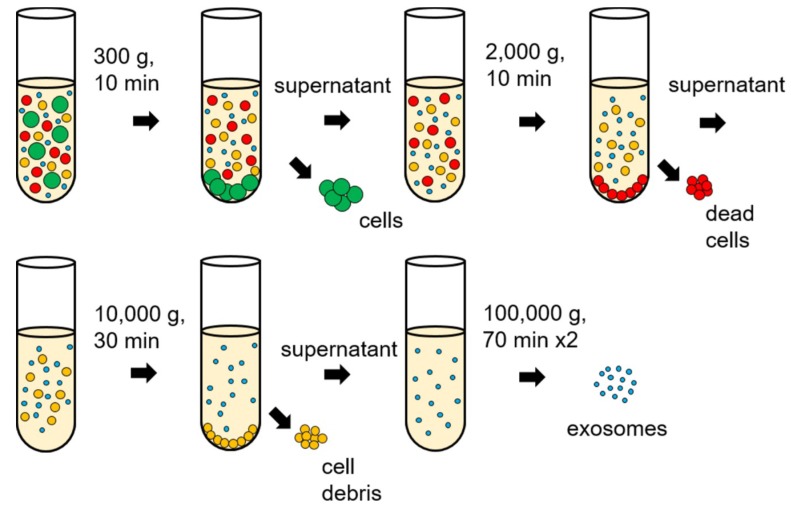
Differential ultracentrifugation process for exosome isolations.

Differential ultracentrifugation is a typical method for exosome separation that uses sequential speed steps of ultracentrifugation (Figure 3). By controlling sequential speeds, the crude homogenate is first centrifuged to sediment cells, dead cells, and cell debris and the resulting pellet is discarded. A classical way to improve exosome purification after differential ultracentrifugation is to isolate the exosome again by ultracentrifugation; in this way, a higher purity is possible, but with a reduced amount of exosome [36,37]. Centrifugation is considered the standard method for separating exosomes from biological fluids/media; however, the major drawback is that the properties of the separated exosomes may depend on the speed of rotation. Moreover, high speeds of rotation can lead to protein aggregation, making results of the Bradford assay less reliable [38]. The efficiency of separation is low and expensive equipment is required to operate differential ultracentrifugation.

### 2.2. Density Gradient Ultracentrifugation

**Figure 4:**
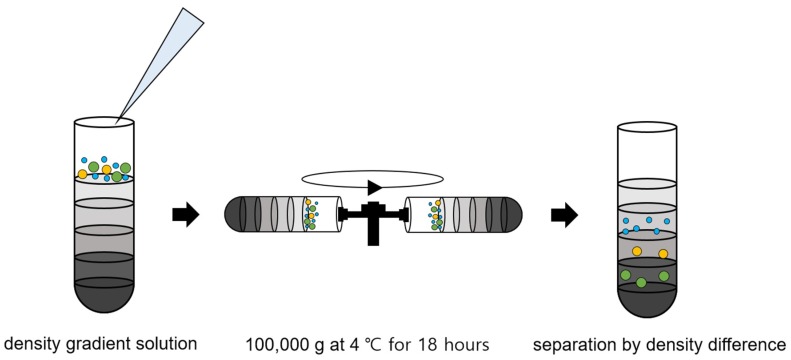
Density gradient ultracentrifugation process for exosome isolation.

Density gradient ultracentrifugation has recently emerged as a technique for sorting exosomes from biological samples. We showed the density gradient ultracentrifugation process in Figure 4. Density gradient ultracentrifugation is a combination of centrifugation and density gradient and used gradient media, such as sucrose, Nycodenz (iohexol), and iodixanol. Exosomes are isolated via ultracentrifugation to the layer in which the density of the gradient media is equal to that of the exosomes [38,39]. This has the advantage of yielding higher purity because the separations are made according to density rather than size. Other advantages include less protein aggregation, less contamination, and good morphological properties. However, to date, this approach has been limited in its reproducibility, since it is very sensitive to the ultracentrifugation time. Moreover, it requires expensive equipment and stacking and selectively separating layers remains difficult.

### 2.3. Size Exclusion

**Figure 5:**
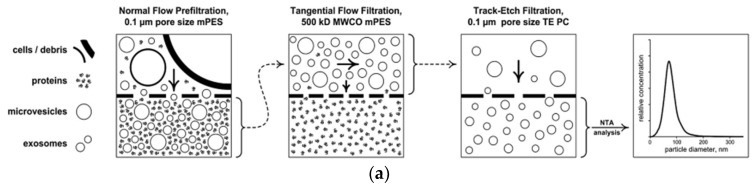

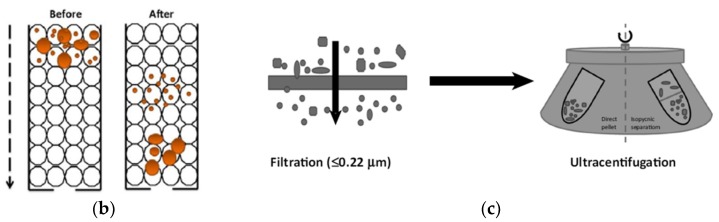
Size exclusion methods. (**a**) filtration [42], (**b**) size exclusion chromatography [46], and (**c**) combination of filtration and ultracentrifugation [45]. Reprinted with permission from [42,45,46].

Figure 5 showed size exclusion methods, including filtration, size exclusion chromatography and the combination of filtration and ultracentrifugation. Exosomes can be purified by size exclusion chromatography (SEC), also referred to as gel permeation chromatography. First, the column is packed with porous polymeric beads with a constant pore size and the separated exosome for diagnosis [40,41,42]. Using an ultrafiltration membrane, exosomes can be separated by size [33,43,44,45,46]. The disadvantage of this method is that the separated exosomes also contain elements of similar size to the exosome such as high density lipoprotein (HDL) or other proteins. Another drawback to this approach is that it is time-consuming and can damage the exosome [47].

### 2.4. Polymer-Based Exosome Isolation

**Figure 6:**
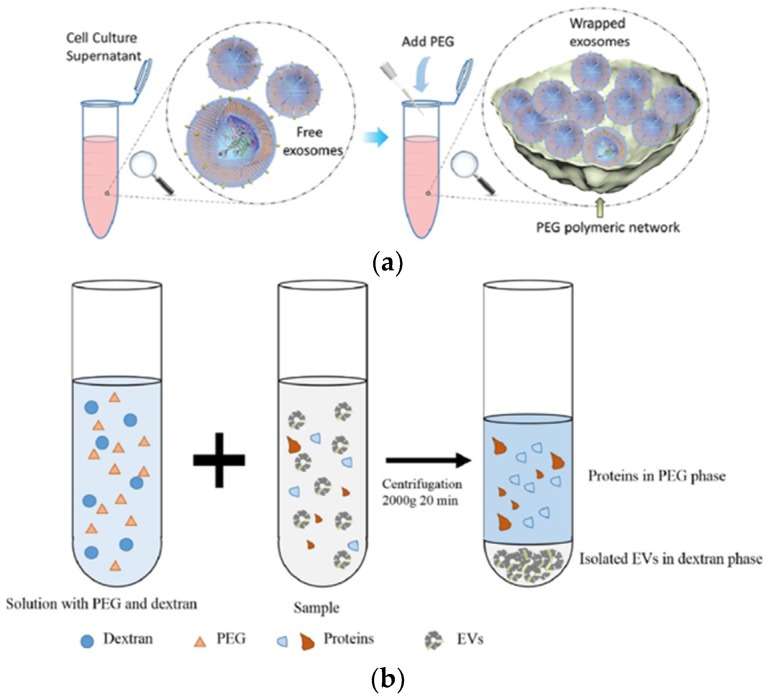
Polymer based exosome isolation of (**a**) polyethylene glycol (PEG) [52] and (**b**) aqueous two-phase system (ATPS) [37]. Reprinted with permission from [37,52].

The polymer-based exosome isolation technique was developed by using polyethylene glycol (PEG) and PEG-dextran-based aqueous two-phase system (ATPS), as shown in Figure 6. For exosome isolation based on PEG, we mixed a biological fluid and a polymer with a precipitating solution, incubated it, and then separated it at low-speed centrifugation (Figure 6a). Generally, Exoquick™ (System Biosciences, USA) is a well-known method for polyethylene glycol (PEG) polymer-based technique [48].

The PEG-dextran-based aqueous two-phase system (ATPS), as shown in Figure 6b, has been suggested for separating particles by a quick and simple process [37,49,50]. Polymer-based exosome precipitation does not require an expensive ultracentrifuge or a large sample volume. The disadvantage of this technique is the low purity of the separated exosome by the inclusion of lipoprotein. In addition, the polymer material can remain in the down-stream analysis [47,51,52].

### 2.5. Immunological Exosome Isolation Techniques

**Figure 7:**
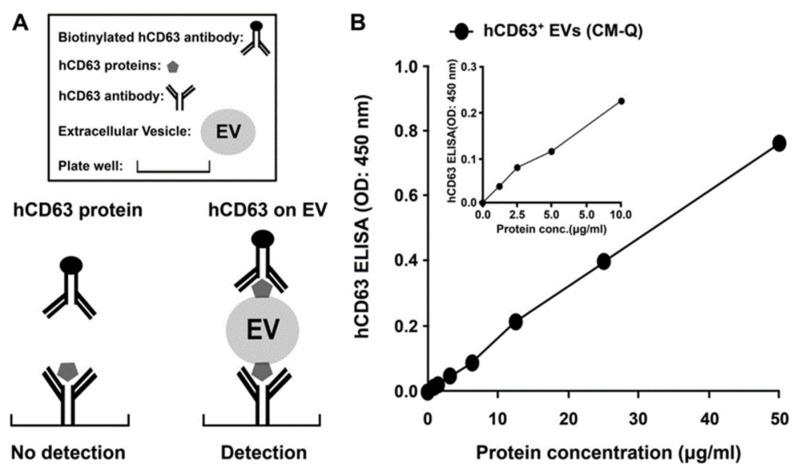
Immunological exosome isolation techniques using enzyme-linked immunosorbent assay (ELISA) [54]. Reprinted with permission from [54].

Immunological methods can selectively isolate exosomes from biological fluids by immobilizing specific capture antibodies that bind to specific proteins on the exosome surface (e.g., CD9, CD81, CD63, HLA-G, and Rab5b) (Figure 7) [36,53,54]. Specific capture antibodies detect CD9, CD63, and CD81 which are known as exosome markers that can be used to isolate exosomes.

Generally, magnetic beads with capture antibodies have been studied [36,53], and enzyme-linked immunosorbent assay (ELISA)-based techniques have also been widely used [55]. ELISA results are expressed as absorbance values which can be used to determine the yield and specificity of the exosome. This approach has several advantages, including its high yield and purity and the selective separation and measurement. The major drawback of this method is that it makes it difficult to handle large volumes, and the isolated antigens lose their activity of the surface functional group [47]. Another major issue is that separation is difficult to achieve in low concentrations because of non-specific binding issues [56].

### 2.6. Exosome Isolation Using Microfluidic Platform

**Figure 8:**
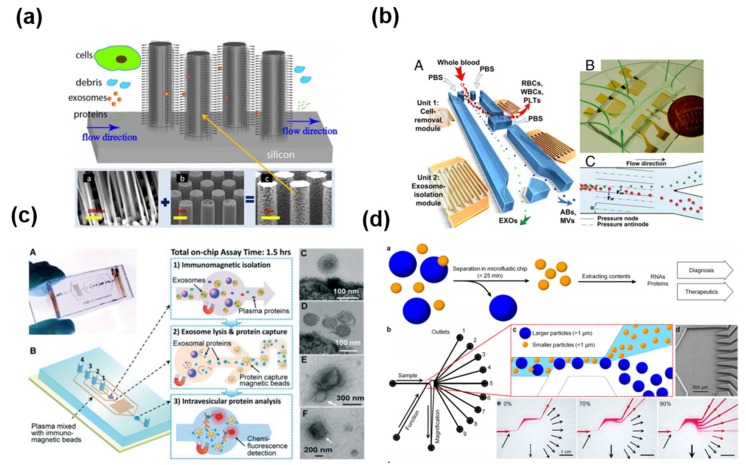
Exosome isolation using microfluidic platform using (**a**) microstructure [57], (**b**) acoustic wave [58], (**c**) immunological separation [60], and (**d**) size based separation [62]. Reprinted with permission from [57,58,60,62].

Microfluidic-based exosome isolation techniques include microstructure-, acoustic wave-, immunological-, and size-based separation (Figure 8). Microfluidics are generally used to separate exosomes by size using micro- or nanostructures (Figure 8a) [57]. Several approaches based on an acoustic wave have been suggested for separating exosomes (Figure 8b) [58]. One interesting way to collect the exosome is through an immunoaffinity technique with a microfluidic channel (Figure 8c) [59,60,61]. In addition, the nanoshearing microfluidics technique with the immunoaffinity method has been reported to increase the reactivity in antibody-antigen interaction. (Figure 8d) [62].

As mentioned above, microfluidics represent a reliable method to separate exosomes according to size, immunoaffinity, and electrical properties. The microfluidic technique is quick, cheap, portable, and easy to automate. Disadvantages include the lack of standardization for clinical samples, limitations of verifying methods, and the small sample volume that can be processed [22].

## 3. Neuronal Exosome-Based Diagnostic Techniques for AD and PD

### 3.1. Exosome Quantification Techniques

**Figure 9:**
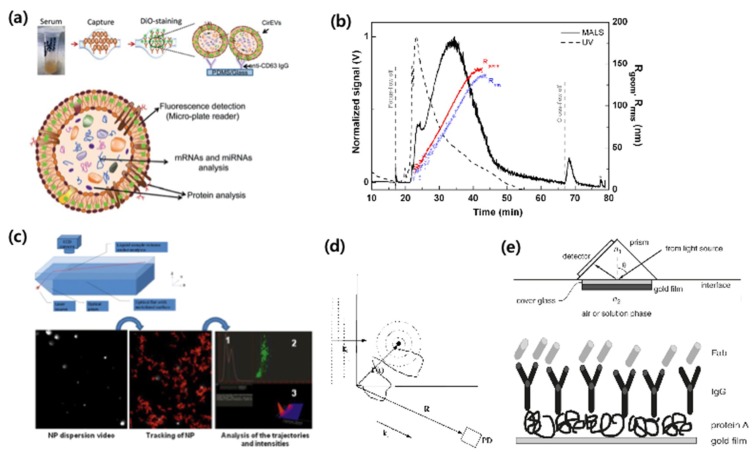
Exosome quantification methods with (**a**) immunoaffinity capture (IAC) [59], (**b**) asymmetrical flow field-flow fractionation (AF4) [63], (**c**) nanoparticle tracking analysis (NTA) [64] (**d**) dynamic light scattering (DLS) [65], and (**e**) surface plasmon resonance (SPR) [66]. Reprinted with permission from [59,63,64,66].

Several exosome quantification techniques have been proposed, as shown in Figure 9.

Immunoaffinity capture (IAC) is the exosome capturing technology via immunoaffinity using an indirect isolation method (Figure 9a) [55,59,67]. In general, using a sandwich assay, IAC enables quantification of exosomes by analyzing color, fluorescence, and electrochemical signals. While it has a high selectivity, its drawbacks are its high cost and long running time.

Asymmetrical flow field-flow fractionation (AF4) is a technique for separating and quantifying molecules using field-flow fraction and diffusion (Figure 9b) [63,67]. This method can separate materials, such as nanoparticles, polymers, proteins, and viruses. Although AF4 is advantageous for its short running time and low cost, it also has a low selectivity.

Nanoparticle tracking analysis (NTA) involves the separation and quantification of particles according to their size (Figure 9c). When the exosome is released from body fluids, the rate of Brownian motion differs according to exosome size. NTA uses the rate of Brownian motion to analyze particles using direct, real-time visualization. This technique also tracks the concentration and size of exosomes using a light-scattering technique [64,68,69]. This means that the measurement cost is relatively low. However, NTA also has a long running time and low selectivity.

Dynamic light scattering (DLS) is a technique that determines particle size by light scattered by particles that exhibit Brownian motion (Figure 9d) [65]. Therefore, DLS can measure exosomes in suspension [65,70,71]. Boyd et al. analyzed the main differences between NTA and DLS, showing give different results, but these are all consistent considering the exact nature of each measure and their physical conditions [72]. A major disadvantage of DLS is that it has a limited selectivity and a long running time. It is also limited in its ability to resolve mixtures of exosomes, because it is biased towards the detection of larger particles [71].

Surface plasmon resonance (SPR) is an immunoaffinity-based assay that captures exosomes with receptors on an SPR sensor surface. When the exosome binds to receptors, its optical signals change, and its resonance can then be quantified through a light source [66,73,74]. SPR has a relatively high selectivity and a short running time, but is costly.

### 3.2. Neuronal Disease-Related Proteins and a New Sensing Platform

Biomarkers of neuronal disease have recently under intense investigation. Aβ, tau, and α-synuclein in neuronal exosomes are great candidates for neuronal disease markers. Specifically, Aβ peptide 42 (Aβ 42), Aβ 40, tau, and tau phosphorylated at threonine 181 (Thr181P) have been extensively studied as biomarkers for AD [75]. However, since they exist in extremely low levels in neuronal exosomes, we described highly sensitive and sensitive sensing platforms below.

**Figure 10:**
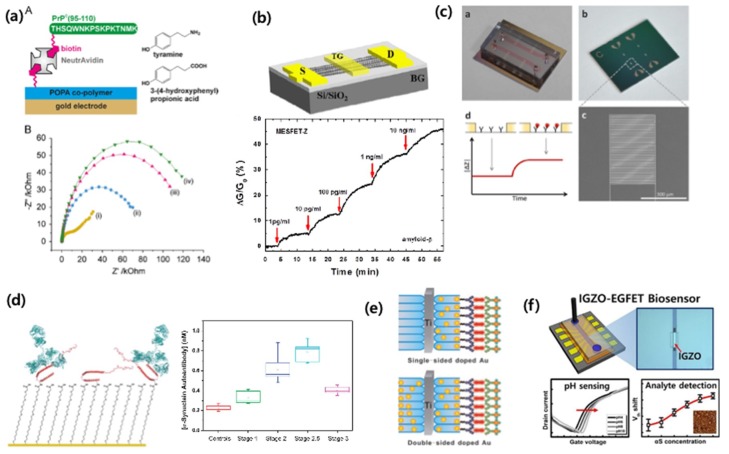
Biomarker detection in exosome for Alzheimer’s disease (AD) and Parkinson’s disease (PD). (**a**) Impedimetric Aβ peptide detection [76]. (**b**) Carbon nanotube-based field effect transistor (FET) for Aβ detection [78]. (**c**) Impediemtric microelectrodes for Aβ detection in mouse plasma [11]. (**d**,**e**) Electrochemical detection of α-synuclein [79,80]. (**f**) electrolyter-gated FET for α-synuclein detection [81]. Reprinted with permission from [11,76,78,79,80,81].

To detect Aβ, Rushworth et al. have developed a method to detect Aβ using electrical impedimetric biosensors (Figure 10a) [76] Rama et al detected Aβ using a cyclic voltammetric immunosensor [77]. Oh et al. developed a biosensor based on a carbon nanotube-based field effect transistor (FET) to measure Aβ in human serum (Figure 10b) [78]. Recently, our group and collaborators developed a microelectrode-based impedimetric biosensor that detects plasma-based Aβ which has high selectivity and sensitivity (Figure 10c) [11,12].

The levels of tau and phosphorylated tau (p-tau) have been reported to be higher in patients with AD [82]. It has been reported that the combined detection of tau, p-tau, Aβ 40, and Aβ 42 CSF levels is more effective than standalone markers in predicting brain Aβ deposition [83].

α-synuclein is known to be associated with PD and rheumatoid dementia. Bryan et al. [79] reported an electrochemical detection-based method for the detection of α-synuclein in human blood (Figure 10d,e) [79]. MasaroeÌk [80] also used electrochemical detection to detect α-synuclein, and Yaruien et al. [84] used nanotubes sensors based on photoelectrochemistry to detect α-synuclein. Our group and collaborators also conducted an amorphous indium gallium zinc oxide (IGZO)-based electrolyte-gated field-effect transistor method to detect α-synuclein, and found that this method had a high selectivity and sensitivity (Figure 10f) [81].

Aβ and tau levels in plasma and serum are much lower than those found in CSF [85]. Considering the lack of sensing ability of commercial assays, detection platforms with a high sensitivity are urgently needed. Using a detection technology with great sensitivity would allow neurodegenerative diseases to be detected from blood-based exosomes.

### 3.3. Detection Techniques using Neuronal Disease-Related Proteins from the Neuronal Exosome

Exosomes have been exploited as a novel source of disease biomarkers, and it has been proposed that exosomes may mediate neurological disease [86]. Exosomal transfer of amyloid into the extra-cellular space could be an important pathway in the development of AD [23]. It has been reported that the neuronal exosome contains Aβ, α-synuclein tau, and microRNA [25,81,87]. Researchers have assessed amyloidopathy and tauopathy and their effects on neurodegenerative disorders.

Aβ deposition in senile plaques and cerebral vessels is a neuropathological feature of AD [88]. Amyloid plaque formation primarily results from Aβ peptides, which are considered to play a pivotal role in AD pathogenesis [89]. Thus, techniques for the detection of Aβ have been intensively studied for the early detection of AD [90].

α-synuclein is a 14-kD amino-acid protein, and the aggregation and dysfunction of α-synuclein are common in neurodegenerative disorders; α-synuclein is closely related to PD and dementia with Lewy bodies [91]. In addition to a neuronal exosome, α-synuclein is an abundant protein in the brain that is easily found in the heart, muscle, and other tissues. It is mainly found in the pre-synaptic terminals of the brain, and interacts with phospholipids and proteins [91].

Tau protein is critically important in axonal maintenance and axonal transport [92]. Total tau (T-tau) and p-tau levels in the brain, blood, and CSF are known to be related to neurodegenerative disorders, and reflect neuronal degeneration; T-tau and p-tau levels are considered to be useful biomarkers for assessing neurodegenerative disorders [93].

ELISA is representative method for Aβ [22,34,87], α-synuclein [25], and tau [22]. Real-time polymerase chain reaction (PCR) has been used to analyze microRNA from neuronal exosomes for the diagnosis of amyotrophic lateral sclerosis (ALS) [22,94,95]. Western blot analysis of α-synuclein has been used to uncover the pathology of PD and rheumatoid dementia [96].

CSF is one of the main biological samples used for exosome detection. Street et al. have reported that ultracentrifugation, Western blot analysis, and transmission electron microscopy can be used to identify and perform proteomic profiling of exosomes in human CSF [97]. Chiasserini et al. showed that exosome in human CSF contain prionogenic proteins such as the amyloid precursor protein and the prion protein [98]. Saman et al have suggested that exosome-mediated secretion of p-tau plays a significant role in the abnormal processing of tau and in the genesis of elevated CSF tau in early AD [99]. Rajendran et al. reported that amyloid peptides are released in association with exosomes [23]. The authors showed that exosomal proteins accumulate in the plaques of human AD brains. 

As mentioned above, CSF collection is an elaborate and complicated process; therefore, the detection of exosomes from human blood is very important in neuronal diagnosis. Hamlett et al. reported that neuronal exosome levels of Aβ 1–42, p-T181-tau, and p-S396-tau were significantly elevated in individuals with Down syndrome compared with age-matched controls [22]. Levels of AD-related proteins were quantified from neuronally derived blood exosomes to identify biomarkers for prediction and staging of mild cognitive impairment and AD [100]. Stern et al. found that tau-positive exosomes in plasma may be a potential biomarker for chronic traumatic encephalopathy [101]. Shi et al. demonstrated the presence of α-synuclein-containing L1 cell adhesion molecule (L1CAM) exosomes in the blood plasma and quantified α-synuclein within these exosomes [24].

## 4. Conclusions and Perspectives

Many reports have suggested that neuronal exosome-based diagnoses can be made from a whole blood; however, the use of exosomes in neuronal disease diagnoses is still in early stages of development. To address these challenges of neuronal exosomal diagnoses, an advanced two-step method for exosome separation and detection is needed.

**Figure 11:**
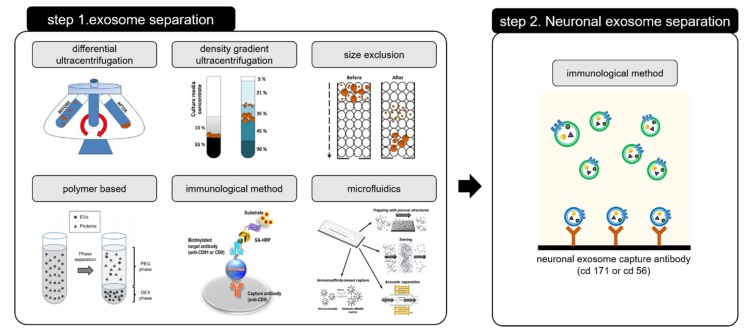
Two-step process requiring for isolating the neuronal exosome. Step 1 for exosome separation [46,49,102] and Step 2 for neuronal exosome separation. Reprinted with permission from [46,49,102].

The two-step method for exosome separation is used to selectively isolate and capture neuronally derived blood exosomes, as shown in Figure 11.

The exosome is first separated from the blood, after which the neuronal exosome is isolated from the separated one. Isolating the neuronal exosome from the blood-based exosome is mainly done by using a specific antigen (CD 56, CD 171) which is bound to the neuronal exosome surface [30,103]. The two-step process for isolating the neuronal exosome is expensive and time consuming. In addition, the loss of the amount of exosome extracted during the two steps results in low efficiency and low yield. Time and cost of exosome separation can be reduced by preventing non-specific binding and improving the reactivity by increased antibody-antigen interactions, making separation simpler. Another approach is to separate/preconcentrate the exosome using a microfluidic platform. We have shown that the use of a microfluidic paper-based analytical device chip with preconcentrating function, yields a 5-fold increase in preconcentrating factors under blood-based biofluids (serum) [104,105]. Based on this technique, our group is now trying to separate and concentrate exosomes simultaneously.

Recent studies have reported that exosomes are related to neurodegenerative disorders; however, one current challenge for the application of exosomes is their isolation method. Nonetheless, exosome-based neuronal diagnostic technologies have a huge potential in the development of clinical devices.
